# Assessing the relationship between nutrition literacy and eating behaviors among nursing students: a cross-sectional study

**DOI:** 10.1186/s12889-023-17468-9

**Published:** 2024-01-02

**Authors:** Pouya Mostafazadeh, Mohammad Javad Jafari, Mohammad Reza Mojebi, Reza Nemati-Vakilabad, Alireza Mirzaei

**Affiliations:** 1 Students Research Committee, School of Nursing and Midwifery, Ardabil University of Medical Sciences, Ardabil, Iran; 2 Student Research Committee, School of Nursing and Midwifery, Guilan University of Medical Sciences, Rasht, Iran; 3 Department of Emergency Nursing, School of Nursing and Midwifery, Ardabil University of Medical Sciences, Ardabil, Iran

**Keywords:** Nutritional literacy, Eating behavior, Nursing students, Nutrition sciences, Iran

## Abstract

**Background:**

Eating behavior is an essential aspect of life that can have long-term effects on health outcomes. Nutrition literacy is crucial for better health and well-being. It empowers individuals to make informed decisions about their nutrition and take control of their eating habits.

**Objectives:**

This study aimed to assess the relationship between nutritional literacy and eating behavior among nursing students at the nursing faculties of Ardabil University of medical sciences.

**Methods:**

A cross-sectional correlational study was conducted in Ardabil province, northwest Iran. The study collected data through simple random sampling at nursing schools in Ardabil province, with 224 nursing students participating. The study collected data from a demographic information form, the nutritional literacy self-assessment questionnaire for students (NL-SF12), and the adult eating behavior questionnaire (AEBQ). The data were analyzed using SPSS version 14.0 software.

**Results:**

Based on the results, nutritional literacy explains 44% of the variance in eating behavior and shows significant explanatory power in two sub-scales of eating behavior. The adjusted R^2^ values for food approach and food avoidance scales were 0.33 and 0.27, respectively.

**Conclusion:**

Given the significant relationship between nutritional literacy and eating behaviors among nursing students, nursing faculty managers and health policymakers should develop new public health strategies to increase nutritional literacy among nursing students.

## Introduction

Nutrition is a crucial aspect of human life, so much so that the World Health Organization (WHO) considers it a significant component of health and development [1]. A well-balanced diet and good nutrition can enhance students’ cognitive performance, intellectual growth, and memory [2]. Research indicates that poor dietary habits during education, particularly at the undergraduate level, can increase students’ risk of chronic diseases [3]. Nursing students, frequently present in clinical settings, are vital in providing nutritional guidance to hospitalized patients and educating them about proper eating behavior [4–6].

Nutrition literacy, which is derived from health literacy, plays a crucial role in determining eating behavior [7]. It refers to understanding and applying healthy nutrition practices [8]. There are various definitions and concepts of nutrition literacy. Still, a comprehensive report describes an individual’s capacity to receive, process, and comprehend essential nutrition information, vital for preventing and managing nutrition-related diseases [9]. Nutrition literacy encompasses six dimensions: knowledge, understanding, obtaining skills, applying skills, interactive skills, and critical skills [10]. Individuals with high levels of nutrition literacy adhere to dietary guidelines to make healthy food choices [11].

Conversely, those with low levels of nutrition literacy may need help with proper nutrition and consequently have poor dietary quality [11]. According to a study conducted by Mengi Çelik & Semerci., which examined the level of nutrition knowledge among nursing students in Turkey, the findings revealed that 91.6% possessed adequate nutrition literacy [4]. Furthermore, another study by Bahramfard et al., which investigated the nutrition literacy status and influencing factors among medical science students in Iran, found that nursing students had sufficient nutrition knowledge but struggled with determining their dietary regimen [12].

Nursing students are regarded as this profession’s young population and future generation [4]. Therefore, they may face challenges such as heavy workloads, long shifts, and intense practical training, which can affect their ability to maintain a proper diet and make them susceptible to various diseases [5]. One necessary solution to address this issue is to enhance the nutrition knowledge of the community, particularly among nursing students [4]. Understanding the factors contributing to a healthy diet and prioritizing nutrition literacy within this group [12]. By increasing nutrition literacy through education, nursing students can gain more control over their dietary choices and opt for healthier options [13].

Nurses have the most interaction and care responsibilities for patients among healthcare professionals, so they spend a significant amount of time promoting health. Compared to other healthcare providers, nurses have higher rates of sedentary behavior, unhealthy diet, overweight, and obesity [14]. The health and well-being of nurses are crucial for healthcare organizations because they directly impact the quality of care [15], patient safety [16, 17], as well as performance and productivity [18]. It is essential for healthcare organizations [19]. The university years are crucial for transforming one’s lifestyle and developing healthy habits, including dietary choices [20]. Therefore, improving healthy eating behavior has been recognized as a critical approach to decreasing the occurrence of non-communicable diseases in both developed and developing countries [21].

Healthy eating behavior is a crucial aspect of life that can have long-term effects on health outcomes [22]. Eating behaviors encompass a range of physiological, psychological, social, and genetic factors that influence meal timing, food intake quantity, food preferences, and food choices [23]. In Iran, a country with an average income, common unhealthy eating behaviors among young people include consuming fast foods and unhealthy snacks, skipping breakfast, and low consumption of fruits, vegetables, whole grains, and dairy products [24]. Among the many factors that affect eating behaviors, nutritional literacy has recently been recognized as a crucial element in enhancing the quality, health, and overall well-being of one’s eating patterns [25]. Consequently, research indicates that different factors, such as eating behaviors, can influence an individual’s level of nutritional literacy [26–29].

Consequently, nursing students must have nutrition-focused courses included in their curriculum. This will help them better understand nutrition, adopt healthier eating behaviors, and prevent diseases [4]. Moreover, promoting healthy eating behaviors among nursing students should also prioritize maintaining physical health, enhancing learning abilities, and supporting academic advancement [25].

The relationship between nutrition literacy and eating behaviors among nursing students in Iran has yet to be extensively studied. However, it is crucial to understand this relationship to encourage healthy eating habits among nursing students and improve their overall health outcomes. Therefore, this study assessed the relationship between nutrition literacy and eating behaviors among nursing students.

## Methods

### Study design and methodology

A cross-sectional correlational study was conducted between February and April 2023 in the Ardabil province of northwest Iran. The study focused on nursing students from three nursing schools - Ardabil, Meshginshahr, and Germi - all affiliated with Ardabil University of Medical Sciences in Iran. The participants were nursing students fluent in Turkish and Persian languages and volunteered to participate in the study. The study excluded individuals with neurological or psychiatric disorders or incomplete data to ensure the accuracy of the results. Additionally, participants who expressed disinterest or were following a special diet were randomly replaced to maintain the integrity of the research. To estimate the sample size, the Epi Info StatCalc program (version 7.0) was used with a confidence level of 95% and a margin of error of 5%. The estimated sample size was 204. To account for a possible non-response rate of 20%, the final sample size was increased to 245 samples.

The researchers contacted the vice-chancellor of Ardabil Midwifery Nursing School to inquire about the number of nursing students in Ardabil province. Subsequently, they determined the number of nursing students in each school. According to their findings, Ardabil Nursing and Midwifery School has 447 nursing students, Germi Nursing School has 147 nursing students, and Meshginshahr Nursing School has 141 nursing students. The researchers used proportional stratified random sampling to determine each faculty’s share in the total sample based on the number of nursing students in each center. They then selected participants using a table of random numbers, taking into account each faculty’s prepared list of nursing students. Participants included first to fourth-year nursing students. The number of participants from each center was predetermined: 149 students from Ardabil Nursing and Midwifery School, 49 from Germi Nursing School, and 47 from Meshginshahr Midwifery Nursing School. Due to data deficiency, the researchers removed 21 incomplete questionnaires (8 from Ardabil, 11 from Germi and two from Meshginshahr) and analyzed data from 224 samples.

### Data collection

#### Demographic information questionnaire

This questionnaire includes questions about demographic characteristics such as age, gender, academic term, height, weight, body mass index (BMI), marital status, level of physical activity, place of residence, frequency of exposure to nutrition-related information at the university (from never to always), perception of personal health status (from poor to don’t know), frequency of eating out (from rarely to 3 or more times a day), smoking history (yes or no), and the name of the nursing school.

#### Nutritional literacy self-assessment questionnaire for students (NL-SF12)

The nutritional literacy self-assessment questionnaire for students (NL-SF12) is a tool developed by Zhang et al. [13]. in 2022 to evaluate students’ cognitive performance and skills related to nutrition. The original form of the questionnaire, NL-43, consists of 43 items. However, the short form of this questionnaire contains 12 questions and six dimensions [30]. These six dimensions include knowledge, understanding, obtaining skills, applying skills, skill application, interactive skills, and critical skills. Knowledge refers to basic nutritional knowledge. Understanding is the ability to read and comprehend nutritional information and recommendations. Obtaining skills is the ability to search for and get nutritional information or services. Skill application refers to applying nutritional knowledge or assistance to maintain a healthy diet. Interactive skills are the ability to interact with food environments that surround us socially and avoid poor eating behaviors or unhealthy food environments. Critical skills are the ability to critically reflect on nutritional information or recommendations based on individual needs. Participants respond to questions using a Likert scale ranging from strongly disagree (1) to agree (5) strongly. In Gao et al.‘s study [30], Cronbach’s alpha for the NL-SF12 tool was reported as 0.89.

After obtaining permission from the tool designer [30], the English version of this questionnaire was translated back and forth, first by two specialized translators independently translating it into Persian without knowledge of each other’s work. Then, both translations were put together, and the best words were selected to create a single version. In the next stage, this Persian text was translated back into English by two translators proficient in English without knowing each other’s work or the original questionnaire text. The translated text was then checked for conformity with the original questionnaire before data collection. To determine content validity ratio (CVR) and relevance ratio, the questionnaire was given to 10 faculty members at Ardabil University of Medical Sciences. The content validity index (CVI) was evaluated separately by experts using three criteria: simplicity, relevance, and clarity on a four-part spectrum (e.g., from very simple to somewhat complex and complex) for each question. Finally, the content validity index and content validity ratio were obtained as 0.91 and 0.88. Additionally, Cronbach’s alpha for the nutritional literacy subscales ranged from 0.73 to 0.89, with an overall nutritional literacy score of 0.84.

This study conducted both Exploratory factor analysis (EFA) and confirmatory factor analysis (CFA) to confirm the factorial structure and the construct validity of the NL-SF12. An exploratory factor analysis with varimax rotation was used to assess the construct validity of the NL-SF12. The results showed that the Kaiser-Meyer-Olkin (KMO) was 0.840, and Bartlett’s test of sphericity was statistically significant (*p* < 0.001, χ^2^ = 1912.335, df = 102), indicating the relevance and appropriateness of the data for conducting the factor analysis. Six factors were extracted that consisted of 12 items and explained 59.57% of the total variance. Also, all items were retained due to the commonalities of < 0.2 and factor loading of < 0.3.

The CFA model was tested using maximum likelihood estimates. The goodness of fit of the model was appraised using multiple criteria including the following: χ^2^/df < 3, Root Mean Square Error of Approximation (RMSEA) < 0.08, Incremental Fit Index (IFI) > 0.90, Normed Fit Index (NFI) > 0.90, Comparative Fit Index (CFI) > 0.90, Goodness of Fit Index (GFI) > 0.90, and Tucker Lewis Index (TLI) > 0.90 [31]. Evaluating the fit of structural equation models: Tests of significance and descriptive goodness-of-fit measures. Methods of psychological research online, 8(2), 23–74.). The goodness-of-fit indices in CFA indicated acceptable values: χ^2^/df = 2.621, RMSEA = 0.059, IFI = 0.967, NFI = 0.948, CFI = 0.957, GFI = 0.926, and TLI = 0.942.

To assess the reliability of the scale, Cronbach’s alpha coefficient (> 0.7) and intraclass correlation coefficient (ICC) (> 0.75) were calculated for the entire scale [32, 33]. The results showed that the NL-SF12 has acceptable reliability. The overall Cronbach’s alpha was 0.86 (knowledge = 0.79, understanding = 0.82, obtaining skills = 0.88, applying skills = 0.83, interactive skills = 0.81, and critical skills = 0.89). The ICC was 0.83 over two weeks.

#### Adult eating behavior questionnaire (AEBQ)

The Adult Eating Behavior Questionnaire (AEBQ) was developed by Hunot et al. in 2016 [34]. The original form of the questionnaire consists of 35 items, which are answered using a 5-point Likert scale ranging from 1 = strongly disagree to 5 = strongly agree. The questionnaire includes two subscales and eight dimensions. The first subscale is the food approach, which consists of four dimensions: enjoyment of food (EF) with three items, emotional over-eating (EOE) with five items, food responsiveness (FR) with three items, and hunger (H) with four items. The second subscale is food avoidance, which includes four dimensions: satiety responsiveness (SR) with three items, emotional under-eating (EUE) with five items, food fussiness (FF) with four items, and slowness in eating (SE) with four items. In 2022, Shamsalinia et al. validated a Persian Version of the Adult Eating Behavior Questionnaire for the first time in Iran [35]. The researchers employed exploratory factor analysis (EFA) and confirmatory factor analysis (CFA) to affirm the ultimate model, which consisted of 31 questions and eight factors [35]. They used various indices, such as RMSEA, PNFI, PCFI, AGFI, GFI, and CMIN/DF, to validate the final model. Impressively, all factors demonstrated acceptable levels of convergent and divergent validity. The study findings revealed that the internal consistency of the eight AEBQ constructs was remarkably high, with a value above 0.8. Additionally, the Composite Reliability (CR) was above 0.7, further supporting the questionnaire’s reliability [35]. The intraclass correlation coefficient (ICC) was determined to be 0.899 (95% CI: 0.917 − 0.878; *p* < 0.001), indicating stability of the AEBQ. Shamsalinia et al. reported a Cronbach’s alpha coefficient of 0.89 for the entire questionnaire, indicating its reliability [35]. In the study, the overall Cronbach’s alpha was 0.73, ranging from 0.71 to 0.86 for the eight dimensions.

### Ethical considerations

This study has been approved by the Ethics Committee of Ardabil University of Medical Sciences with the ethics code (NO: IR.ARUMS.REC.1401.277). The study objectives were explained to each participant at the beginning of the study, and they were given the right to withdraw from the study at any time. Participation in the survey was voluntary for all individuals. Written informed consent was obtained from nursing students. All stages of this study were conducted by the Helsinki Declaration. Ethical considerations such as confidentiality, anonymity, and keeping information confidential were observed in this study.

### Statistical analysis

The statistical package for social sciences (SPSS) version 14.0 and IBM AMOS 20.0 was used for statistical analysis and validation of results. Percentage, frequency, mean, standard deviation, and a confidence level of 0.95 were used to describe study variables. Pearson’s correlation coefficient, Independent-sample *t*-test, and one-way ANOVA were used to determine the relationship between nutrition literacy level and eating behaviors with demographic variables. Hierarchical regression was used to predict eating behavior and its subscales. A *p*-value ≤ 0.05 was considered statistically significant, and a *p*-value ≤ 0.001 was considered highly significant.

## Results

Two hundred twenty-four nursing students (98 males, 126 females) participated in the study. The mean age of participants was 22.8 ± 3.16 years, and the mean BMI was 22.70 ± 3.22 kg/m^2^. 89.7% of the students were single. The majority of participants (58%) reported moderate physical activity levels. Most participants resided in urban areas and described their health status as good. 33.9% of students had never been exposed to nutritional information, while 46.9% used outside food sources 1–3 times per week. Only 25.4% had a history of smoking. Demographic characteristics of nursing students are presented in Table 1.

**Table 1:** Demographic characteristics of the participants (*N* = 224)

Variables	Mean	SD
**Age**	22.08	3.163
**BMI**	22.70	3.228
	**n**	**%**
**Semester**		
First semester	12	5.4
Second semester	38	17.0
Third semester	31	13.8
Fourth semester	48	21.4
Fifth semester	62	27.7
Sixth semester	33	14.7
**Gender**		
Male	98	43.8
Female	126	56.3
**Marital status**		
Single	201	89.7
Married	23	10.3
**Physical activity level**		
Physical inactive	39	17.4
Moderate activity level	130	58.0
Regular activity level	42	18.8
Regular extensive activity	13	5.8
**Residence**		
village	15	6.7
countryside	24	10.7
City center	185	82.6
**Frequency of exposer to Nutrition-related information at university**		
Never	76	33.9
seldom	57	25.4
sometimes	72	32.1
often	3	1.3
always	16	7.1
**The level of perception of your health**		
Poor	15	6.7
moderate	76	33.9
Good	129	57.6
I do not know	4	1.8
**Frequency of eating out**		
Seldom	60	26.8
1–3 meals per weeks	105	46.9
4–6 meals per weeks	33	14.7
1–2 meals daily	19	8.5
3 or more meals daily	7	3.1
**Smoking history**		
*Yes*	57	25.4
*No*	167	74.6
**The name of the nursing faculty**		
Ardabil	141	62.9
Germi	38	17.0
Meshginshahr	45	20.1

The level of nutrition literacy among nursing students is presented in Table 2. The mean score on the nutrition literacy questionnaire was 3.37 (95% CI: 3.28 to 3.46). Among the dimensions of nutrition literacy, the highest score was related to knowledge, 3.60 (95% CI: 3.43 to 3.77) and interactive skills, 3.50 (95% CI: 3.36 to 3.62). In contrast, the lowest score was related to applying skills 2.90 (95% CI: 2.85 to 3.13) and obtaining skills 3.25 (95% CI: 3.13 to 3.37).

**Table 2:** The status of nutrition literacy among nursing students (*N* = 224)

Items	Mean	SD	(95% CI)	
**Knowledge**	**3.60**	**1.304**	**3.43**	**3.77**
1. Balanced diet and reasonable nutrition are important measures to prevent and control chronic diseases such as diabetes and hypertension.	3.59	1.469		
2. Steaming and boiling are healthier ways of cooking than frying and grilling.	3.61	1.354		
**Understanding**	**3.40**	**0.903**	**3.27**	**3.51**
3. I can easily understand the nutritional information delivered by new and traditional media.	3.37	1.076		
4. I have a good understanding of expert consensus regarding nutrition or dietary information.	3.43	1.056		
**Obtaining skills**	**3.25**	**0.941**	**3.13**	**3.37**
5. I know where to find healthy diet information.	3.34	1.097		
6. I often read nutrition information transmitted through new media (e.g., WeChat and microblogging) or watch nutrition-related program	3.16	1.208		
**Applying skills**	**2.99**	**1.041**	**2.85**	**3.13**
7. I drink milk or dairy products every day.	3.02	1.287		
8. I often buy foods based on nutrition facts on food packages.	2.95	1.320		
**Interactive skills**	**3.50**	**1.005**	**3.36**	**3.62**
9. I am open to reasonable nutrition and health advice from family or friends.	3.52	1.182		
10. If my family members or friends are overweight and enjoy eating high-fat foods, I will encourage them to make dietary changes.	3.47	1.298		
**Critical skills**	**3.48**	**0.984**	**3.36**	**3.60**
11. I can easily tell whether my daily diet is reasonable.	3.50	1.171		
12. I can estimate the suitable food intake for maintaining a healthy body weight.	3.45	1.178		
**Total**	**3.37**	**0.735**	**3.28**	**3.46**

Abbreviation: CI, confidence interval

The mean score on the eating behavior questionnaire was 3.18 (95% CI: 3.12 to 3.23) (Table 3). Among the dimensions of the “food approach” scales, the highest score was related to the enjoyment of food, 3.84 (95% CI: 3.72 to 3.94), and the lowest score was related to emotional over-eating 2.82 (95% CI: 2.72 to 2.92). Among the dimensions of the “food avoidance” scale, the highest score was related to food fussiness, 3.48 (95%CI: 3.41 to 3.56), and the lowest score was related to slowness in eating, 3.08 (95% CI: 2.99 to 3.17).

**Table 3:** The status of eating behaviors among nursing students (*N* = 242)

Items		Mean	SD	(95% CI)	
**Enjoyment Food**		**3.84**	**0.846**	**3.72**	**3.94**
EF 1	I love food	4.01	1.024		
EF 3	I enjoy eating	4.12	0.930		
EF 4	I look forward to mealtimes	3.38	1.134		
**Emotional Over-Eating**		**2.82**	**0.819**	**2.72**	**2.92**
EOE 5	I eat more When I’m annoyed	2.70	1.360		
EOE 8	I eat more When I’m worried	3.38	1.317		
EOE 10	I eat more When I’m upset	2.64	1.354		
EOE 16	I eat more When I’m anxious	2.66	1.291		
EOE 21	I eat more When I’m angry	2.73	1.287		
**Emotional Under-Eating**		**3.18**	**0.666**	**3.10**	**3.27**
EUE 15	I eat less When I’m worried	3.33	1.223		
EUE 18	I eat less When I’m angry	3.28	1.279		
EUE 20	I eat less When I’m upset	3.41	1.242		
EUE 27	I eat less When I’m annoyed	3.35	1.222		
EUE 35	I eat less When I’m anxious	2.56	1.240		
**Food Fussiness**		**3.48**	**0.591**	**3.41**	**3.56**
FF 7	I refuse new foods at first	2.74	1.257		
FF 12	I enjoy tasting new foods	3.71	1.062		
FF 19	I am interested in tasting new food I haven’t tasted before	3.75	0.988		
FF 24	I enjoy a wide variety of foods	3.74	1.084		
**Food Responsiveness**		**2.87**	**0.849**	**2.76**	**2.99**
FR 13	I often feel hungry when I’m with someone who is eating	3.28	1.170		
FR 17	Given the choice, I would eat most if the time	2.65	1.117		
FR 22	I am always thinking about food	2.69	1.189		
**Hunger**		**3.13**	**0.832**	**3.02**	**3.23**
H 9	If I miss a meal, I get irritable	3.21	1.245		
H 28	I often feel so hungry that I have to eat something right away	3.09	1.130		
H 32	I often feel hungry	2.95	1.029		
H 34	If my meals are delayed, I get light-headed	3.26	1.170		
**Slowness In Eating**		**3.08**	**0.654**	**2.99**	**3.17**
SE 14	I often finish my meals quickly	3.19	1.306		
SE 25	I am often last at finishing a meal	3.02	1.350		
SE 26	I eat more and more slowly during the course of a meal	3.08	1.297		
SE 29	I eat slowly	3.04	1.290		
**Satiety Responsiveness**		**3.02**	**0.825**	**2.91**	**3.13**
SR 23	I often get full before my meal is finished	2.79	1.213		
SR 30	I cannot eat a meal if I have had a snack just before	3.2009	1.075		
SR 31	I get full up easily	3.06	1.002		
**Food Approach**		**3.17**	**0.612**	**3.09**	**3.24**
**Food Avoidance**		**3.19**	**0.001**	**3.14**	**3.25**
**Total**		**3.18**	**0.398**	**3.12**	**3.23**

Abbreviation: CI, confidence interval

The level of nutrition literacy and eating behavior of individuals based on demographic characteristics are presented in Table 4. Nutrition literacy significantly correlated with the frequency of exposure to nutrition-related information at the university and college. Additionally, the status of eating behavior had a significant negative correlation with BMI and a significant positive correlation with physical activity level (*p* < 0.05).

**Table 4:** Relationship between nursing students’ general characteristics with nutrition literacy and eating behavior (*N* = 224)

Variables	Nutrition literacy			Eating behavior		
	Mean	SD	*p*-value	Mean	SD	*p*-value
**Age**	3.37	0.735	**r = -0.035** ***p*** **= 0.598**	3.1835	0.39816	**r = − 0.062** ***p*** **= 0.356**
**BMI**	3.37	0.735	**r = -0.062** ***p*** **= 0 0.356**	3.1835	0.39816	**r = -0.163** ***p*** **= 0.015**
**Semester**			**F = 1.610** ***p*** **= 0.159**			**F = 0.505** ***p*** **= 0.772**
First Semester	2.84	0.768		3.12	0.215	
Second Semester	3.33	0.755		3.21	0.550	
Third Semester	3.44	0.792		3.17	0.403	
Fourth Semester	3.34	0.717		3.17	0.325	
Fifth Semester	3.46	0.687		3.22	0.405	
Sixth Semester	3.41	0.723		3.10	0.324	
**Gender**			**t = -0.239** ***p*** **= 0.811**			**t = 3.459** ***p*** **= 0.107**
Male	3.36	0.719		3.13	0.449	
Female	3.38	0.749		3.22	0.350	
**Marital status**			**t = 1.279** ***p*** **= 0.202**			**t = 1.207** ***p*** **= 0.229**
Single	3.39	0.722		3.19	0.403	
Married	3.18	0.836		3.08	0.345	
**Physical activity level**			**F = 2.001** ***p*** **= 0.115**			**F = 3.633** ***p*** **= 0.014**
Physical inactive	3.21	0.723		3.01	0.310	
Moderate activity level	3.36	0.729		3.20	0.373	
Regular activity level	3.42	0.754		3.20	0.387	
Regular extensive activity	3.77	0.677		3.38	0.701	
**Residence**			**F = 1.048** ***p*** **= 0.352**			**F = 0.595** ***p*** **= 0.552**
village	3.55	0.801		3.17	0.452	
countryside	3.21	0.818		3.10	0.295	
City center	3.38	0.718		3.19	0.405	
**Frequency of exposer to Nutrition-related information at university**			**F = 2.382** ***p*** **= 0.050**			**F = 0.629** ***p*** **= 0.643**
Never	3.27	0.797		3.14	0.397	
seldom	3.57	0.629		3.22	0.433	
sometimes	3.36	0.741		3.20	0.377	
often	3.91	0.901		3.30	0.246	
always	3.10	0.576		3.10	0.394	
**The level of perception of your health**			**F = 2.195** ***p*** **= 0.090**			**F = 0.924** ***p*** **= 0.430**
Poor	3.02	0.583		3.06	0.245	
moderate	3.28	0.704		3.21	0.334	
Good	3.46	0.760		3.18	0.431	
I do not know	3.50	0.619		2.99	0.778	
**Frequency of eating out**			**F = 0.873** ***p*** **= 0.481**			**F = 1.572** ***p*** **= 0.183**
Seldom	3.41	0.732		3.17	0.412	
1–3 meals per weeks	3.31	0.719		3.14	0.357	
4–6 meals per weeks	3.34	0.744		3.17	0.441	
1–2 meals daily	3.45	0.875		3.31	0.495	
3 or more meals daily	3.80	0.519		3.45	0.258	
**Smoking history**			**t = -0.447** ***p*** **= 0.656**			**t = -0.983** ***p*** **= 0.327**
Yes	3.33	0.718		3.13	0.371	
No	3.38	0.742		3.19	0.406	
**The name of the nursing faculty**			**F = 3.080** ***p*** **= 0.048**			**F = 0.257** ***p*** **= 0.773**
Ardabil	3.46	0.700		3.16	0.405	
Germi	3.27	0.752		3.21	0.422	
Meshginshahr	3.17	0.792		3.20	0.357	

This study used nutritional literacy to predict eating behavior and its subscales. Our results showed that the final stage of the hierarchical regression model for eating behavior was significant, F (13.066), *p* < 0.001, adjusted R^2^ = 0.41 (Table 5). Based on the results, nutritional literacy explained 44% of the variance in eating behavior and demonstrated significant explanatory power in two eating behavior subscales. The adjusted R^2^ values for the food approach and food avoidance subscales were 0.27 (*p* < 0.001) and 0.33 (*p* < 0.001), respectively.

**Table 5:** Hierarchical regression model for eating behavior explained by nutrition literacy (Model 2)

Predictors	Eating behavior	Food approach	Food avoidance
Β	0.62***	0.56***	0.33***
∆ *R*^2^	0.44	0.37	0.31
∆*F*	134.095***	97.757***	31.783***
Adjusted *R*^2^ (Final)	0.41	0.33	0.27
*F* (Final)	13.066***	9.520***	7.562***
*Df*_1_, *Df*_2_	1,210	1,210	1,210

Model 1 controls for age, BMI, Seamaster, gender, marital status, physical activity level, residence, frequency of exposure to nutrition-related information at university, the level of perception of your health, frequency of eating out, and smoking history. ***p < 0.001

## Discussion

According to our knowledge, this was the first study to examine the relationship between nutrition literacy and eating behaviors in college students, including eating approach and avoidance behaviors. Li and colleagues found a positive correlation between nutrition literacy scores and healthy eating behaviors in nursing students, with nutrition literacy being a strong predictor of eating approach and avoidance behaviors [28]. Siow et al. also found a relationship between nutrition literacy and adult eating behaviours. Additionally, students with a plan for healthy eating had higher nutrition literacy scores and healthier eating behaviors than their peers [8]. This suggests that individuals who prioritize a healthy lifestyle can improve and maintain their eating behaviors. Therefore, nutrition literacy interventions that include developing a healthy eating plan can improve eating behaviors [8]. Studies have also reported that individuals’ eating behavior is influenced by their nutrition literacy levels [27], and healthy eating behaviors are positively associated with NL skills [36, 37]. Prioritizing personal health has a positive impact on eating habits. By increasing one’s nutrition knowledge and creating healthy meal plans, individuals can effectively influence their eating behavior and improve their overall health and well-being. Nutrition education plays a significant role in promoting healthy eating habits; therefore, it is crucial to prioritize nutrition literacy.

In our study, there was a relationship between eating behavior and physical activity level. According to Farahani’s study, unhealthy behaviors such as eating behaviors are associated with insufficient physical activity and increased sedentary behavior [38]. Another survey on adolescents showed a significant relationship between their friends’ eating behavior and their BMI because they usually have similar eating behaviors, making them somewhat identical in weight status [38].

Evidence shows positive progress in eating behavior accompanied by increased physical activity. Individuals who were engaged in sports showed healthier eating behaviors. This study observed physical activity as a determinant of eating behavior. Students who did not engage in physical activity lacked motivation to consume a balanced diet [37]. This analysis is consistent with a study by Downes et al., which stated that physical activity is a strong motivator for healthy eating behaviors among students [39]. Shinde et al.‘s study on Indian healthcare professionals showed a significant positive correlation between the eating behavior scale and BMI. This study showed that eating behavior was associated with obesity, daily physical activity, or sedentary lifestyle [40].

Based on the findings of this study, the mean score for nutritional literacy among nursing students was 3.37 out of 5, indicating that they had sufficient nutritional literacy. In Bahramfard et al. study on medical students in Iran who used the Adult Nutrition Literacy Assessment (EINLA) tool, the total score for nutritional literacy was reported as 24.92 out of 35 [12]. In Lai et al. study on Taiwanese students who used the Nutrition Literacy Scale (NL scale), the total score for nutritional literacy was 4.32 out of 6 [20]. However, in a study conducted by Siow et al., on adults in Malaysia who used the self-rated nutrition literacy scale, the mean nutrition literacy score was reported as 17.66 out of 30. Additionally, 80% of participants in this study were found to have poor nutrition literacy [41]. In Ashoori et al., on Iranian youth who used our original questionnaire form, the average nutritional literacy score was 52.1 out of 100, indicating low levels of nutritional literacy among Iranian youth and highlighting the need to improve their nutritional literacy skills [24]. The difference in nutrition literacy levels may be due to limited exposure to nutrition information among certain population groups, differences in individuals’ education levels, societal pressures and norms, or socio-economic status.

Among the dimensions of nutrition literacy, the highest score was related to the knowledge dimension, which was consistent with the findings of Liao et al. This study showed that because most students have access to the internet and can search for and obtain nutrition information on social media platforms, they have acquired a high level of knowledge about nutrition [28]. Nowadays, social media platforms are becoming one of the sources of nutrition and health information for students. However, in our study, students obtained the lowest score in nutrition literacy skill application dimension [37]. This result may be due to the ease of access to a large volume of health and nutrition-related content today, causing students to need clarification when selecting accurate and precise information and applying it effectively [24, 41].

The study results showed that the mean overall score of students’ eating behavior was 3.18 out of 5. This result was consistent with Lee C-K et al. and Liday DM et al. studies [8, 42]. but did not match Liao and colleagues’ findings. In Lee et al.‘s study, the mean overall score of students’ eating behavior was 39.97 out of 65 [24], while in the Liday DM study, it was reported as 2.62 out of 4 [42]. Mamun et al. considered good eating behavior to result from high awareness about health status, extensive knowledge about nutrition, and a positive attitude towards healthy food [43]. Liao et al.‘s findings indicated that students’ eating behavior was unsatisfactory [28]. Additionally, Siow et al.‘s study on adults found that the mean overall score of eating behavior was 88.26 out of 52, indicating that most respondents (74.5%) had poor eating behavior due to the prevalence of low-quality diets, increased frequency of eating outside the home, and consumption of low-quality and harmful food [41].

Food enjoyment received the highest score among the dimensions of the scale (food approach). This finding was consistent with Hunot-Alexander et al. [44] and He J et al. [45] studies. Nowadays, due to the availability of restaurants, fast foods, and other food-related stores along with media influence, encouraging students to consume food as a form of recreation and enjoyment [46]. Emotional Over-Eating received the lowest score among the scale dimensions (food approach). Previous studies conducted by Zickgraf et al. on candidates for bariatric surgery in America [47] and Dubois et al. study [48] also found that emotional over-eating received the highest score. Due to various psychological workshops for students, such as communication skills and prevention of emotional relationship damage, nursing students learn how to manage their emotions and feelings well in crises, which is why they are less prone to emotional overeating.

Among the dimensions of the scale (food avoidance), the highest score was related to the dimension of food fussiness, consistent with a study conducted by Shamsalinia on epileptic patients [35]. Considering that the type of food is rooted in the culture of that geographical region and the available food ingredients and that most students are non-native and live independently away from their families, they may experience food confusion. Bookari et al. showed that parental eating habits and nutrition strategies are the most influential factors in determining eating behavior and food choices. Parents actively choose what their family eats, act as role models for food choices and patterns, and use eating practices to reinforce preferred eating patterns and behaviors [49]. Another study on adolescents reported that regular family meals might promote healthy eating behaviors and serve as models for healthy food choices. Family structure and socioeconomic status have also been identified as determinants of nutritional status [24, 50]. However, given that in Iran, non-native students are mostly accepted in universities located in cities similar in terms of food culture, they are open to trying new foods from that region and show a willingness to do so.

The results showed that the lowest score among the scale dimensions (food avoidance) was related to the slowness in the eating dimension, which is consistent with the results of a study conducted by Alruwaitaa et al. on adults [51]. It can be said that due to the heavy workload of university, students may have limited time or interest in developing their food and nutrition-related skills, such as eating, shopping, preparing and cooking food (performance skills) or interacting with others about food and nutrition (interactive skills) [24]. According to previous studies, it can be interpreted that nursing students may need more time to eat due to heavy university curricula and hospital internships, which is why they are less likely to experience difficulties in eating.

This study showed a significant relationship between nutrition literacy and students’ exposure to nutrition-related information in universities and places of study. This finding is consistent with previous studies [52, 53] and suggests that individuals who receive nutrition information in universities and have completed relevant nutrition courses have better nutrition literacy [52]. Additionally, using food and nutrition information obtained through various media channels may be challenging for university students due to the university’s unique environment [30]. Insufficient nutrition literacy among students who do not attend central provincial universities may be due to underdeveloped economic conditions, low income, poor quality of life, limited access to nutrition information, and low awareness of eating behaviors [54, 55]. Therefore, an inappropriate learning environment affects the nutrition literacy of students and leads to unhealthy eating behaviors [49, 55]. Alotaibi et al., who examined the geographical impact on nutrition literacy levels and eating behaviors in the United States, found that nutrition literacy and eating behaviors vary depending on individuals’ residential areas [50].

One of the findings of the present study was that there was a significant relationship between eating behavior and BMI. Herle et al. also found similar results to ours, stating that there is a significant correlation between BMI and eating behaviors [55]. Individuals with unhealthy eating behaviors are likely to have a higher BMI. Therefore, individuals with a healthy diet believe it helps them maintain their body weight within a healthy range as much as possible [56]. However, Natour et al.‘s results contradicted ours, stating that individuals with higher BMI are more likely to engage in healthier behaviors. In comparison, those with lower BMI are less likely to engage in healthy behaviors [55].

Our study differed from previous ones in two ways. Firstly, we assessed the correlation between nutritional literacy and eating behaviors, in nursing students rather than the general population. Secondly, we explored the relationship between healthy behavior and physical activity levels in nursing students. We used standardized instruments to measure nutritional awareness and behaviors, which can lead to a better understanding of dietary behaviors in nursing students.

Nursing students often experience changes in their eating behaviors due to their entry into independent lifestyles, and can easily exhibit unhealthy eating behaviors. Unhealthy eating behaviors can lead to illness and consequently reduce students’ daily and academic quality of life. Nutritional literacy is an essential factor in determining eating behavior and plays a crucial role in improving the health and well-being of students. According to our knowledge, this is the first study to examine the relationship between nutritional literacy and eating behaviors among nursing students in Iran, which can reflect the weaknesses of current nursing curricula in improving nutritional literacy and nutrition among students.

### Limitations

This study was subject to the following limitations: firstly, the cross-sectional nature of this study does not allow for causal interpretations. Secondly, this study was conducted only in nursing faculties of Ardabil University of Medical Sciences and had a small sample size, therefore the findings cannot be generalized across the country. Thirdly, since the tools used in this research were self-report questionnaires, personal mental feelings may have been inconsistent with actual behaviors and may have caused errors in data collection. Finally, our aim was to investigate the relationship between nutritional behaviors and nutritional literacy among nursing students. Therefore, we did not add factors affecting nutritional knowledge to the questionnaire. It is recommended that future studies examine factors affecting the level of nutritional knowledge among nursing students.

## Conclusion

The mean score on the nutrition literacy questionnaire was 3.37. Among the dimensions of nutrition literacy, the highest score was related to knowledge, 3.60 and interactive skills, 3.50. In contrast, the lowest score was related to applying skills 2.90 and obtaining skills 3.25. The mean score on the eating behavior questionnaire was 3.18. Among the dimensions of the “food approach” scales, the highest score was related to the enjoyment of food, 3.84 and the lowest score was related to emotional over-eating 2.82. Among the dimensions of the “food avoidance” scale, the highest score was related to food fussiness, 3.48, and the lowest score was related to slowness in eating, 3.08. According to the results, nutrition literacy explains 44% of variance in nutrition behavior and shows significant explanatory power in two sub-scales of nutrition behavior. The adjusted R^2^ values for food approach and food avoidance scales were (*p* < 0.001), 0.27 (*p* < 0.001), and 0.33 respectively. Nutrition literacy as a combination of cognitive and behavioral knowledge and skills has potential for addressing weaknesses in eating behaviors and improving healthy decision-making about eating behaviors. Given the direct relationship between nutrition literacy and eating behaviors, nursing school administrators and health policymakers should develop new public health strategies focused on increasing nutrition literacy among nursing students. Quantitative studies have evaluated the relationship between nutrition literacy and eating behaviors among nursing students using healthy eating behavior questionnaires and original forms of nutrition literacy assessments; however, these studies are not easily comparable. Therefore, conducting further studies using a short form of nutrition literacy questionnaire and similar eating behaviors as we used to be highly recommended.

## Data Availability

The data that support the findings of this study are available from the corresponding author [A.M.] upon request.
